# Proposal of statistical twin as a transition to full digital twin technology for cardiovascular interventions

**DOI:** 10.1093/icvts/ivae032

**Published:** 2024-03-04

**Authors:** Peyman Sardari Nia, Yuri Ganushchak, Jos Maessen

**Affiliations:** Department of Cardiothoracic Surgery, Maastricht University Medical Centre, Maastricht, the Netherlands; Foundation Heart Team Academy, Maastricht, the Netherlands; Department of Cardiothoracic Surgery, Maastricht University Medical Centre, Maastricht, the Netherlands; Foundation Heart Team Academy, Maastricht, the Netherlands; Department of Cardiothoracic Surgery, Maastricht University Medical Centre, Maastricht, the Netherlands; Foundation Heart Team Academy, Maastricht, the Netherlands

**Keywords:** Statistical twin, Digital twin, Predictive models, Risk scoring

## Abstract

**OBJECTIVES:**

We introduced statistical twin as aggregates of multiple virtual patients’ data throughout the treatment at any chosen time point. The goal of this manuscript was to provide the proof of concept of statistical twin by evaluating the feasibility of detection of distinctive aggregates of patients throughout the perioperative trajectory (prerequisite for development of statistical twin).

**METHODS:**

We used a retrospective validated cohort of all comers with mitral valve disease treated (2014–2020) at a tertiary academic hospital. The end point was overall survival based on the decision of the heart team. We applied two-step cluster analysis to detect distinct aggregated virtual patients throughout the process of care.

**RESULTS:**

The cluster procedure resulted in 5 distant clusters with relatively equal numbers of patients. Effects of the treatment (surgery, transcatheter or optimal medical therapy) on survival were as follows: For optimal medical therapy, the expected survival ranged from 95% to 96% in 30 days to 58% to 75% in 10 years independent of baseline characteristics. However, for transcatheter interventions, the 5-year survival was 60–92% and was dependant on the initial characteristics of the virtual patient. Furthermore, survival following an uncomplicated operation of normal duration was higher through all observation periods. The aggregated virtual patients of cluster 5 would have a better survival rate at all times if the intervention were done by a dedicated surgeon.

**CONCLUSIONS:**

It is possible to detect distinctive aggregates of virtual patients based on baseline characteristics and to capture the impact of perioperative events and external and other factors at multiple time points throughout the postoperative phase.

**Figure:**
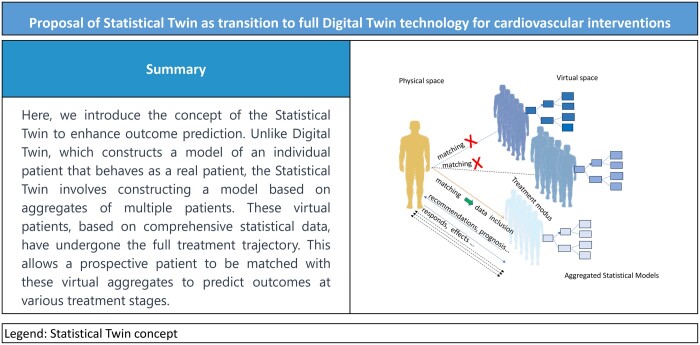

## INTRODUCTION

The guiding principle in the allocation of treatment for cardiovascular interventions is risk assessment, which is based primarily on the European System for Cardiac Operative Risk Evaluation (EuroSCORE) and the Society of Thoracic Surgeons (STS) score [1, 2]. Unfortunately, both the EuroSCORE and the STS scores focus primarily on 30-day mortality, overlooking the prediction of morbidity in the postoperative period and failing to predict individual long-term outcomes. Additionally, the predictions made by these risk scoring systems are based on preoperative constants, including the planned intervention, and do not take into account perioperative events that determine short-term and long-term outcomes.

Development of the human digital twin (DT) is the ideal model for predicting the patient’s trajectory through process of care [3]. The DT is a model of the object, consisting of an evolving set of data related to the object, along with a means of dynamically updating or adjusting the model in accordance with these data. Therefore, in DT technology, the model behaves exactly like a real patient at any given time point, subject to any induced variable. The clinical implementation of digital twins requires solving a wide range of technical, medical, ethical, and theoretical challenges [3]. Introduction of DT in clinical practice offers predictive abilities beyond the traditional “predictor” technologies. This DT paradigm is an ultimate departure from traditional big data statistical techniques like logistic regressions. The DT systems generate virtual twins via deep phenotyping [4]. The physical twin data can be used to measure and then forecast patient response to medication, behaviour change and environmental factors [5]. However, there is a long way to go, not only because of its extreme complexity, which requires the development of related technologies such as data mining, data fusion analysis, artificial intelligence, deep learning and human computer science, but also because so many aspects are involved, including the security and social ethics problems [6].

Development of “virtual patients” as larger aggregates of the data of multiple patients could be a transitional step in departure from traditional big data statistical techniques to individual deep phenotyping. Detection of distinctive groups of patients based on the demographic and clinical data allows forecasting with less variability using the patients’ responses to the treatment, thereby enhanceing selection of the most appropriate ‘individual’ method of treatment.

To enhance the predictive value of the traditional risk assessment system, we propose and introduce the concept of a statistical twin. Unlike the DT approach, which constructs a model of an individual patient, the statistical twin approach involves constructing a model based on aggregates of multiple patients. These virtual patients, based on statistical data, have undergone the full treatment trajectory. This system allows a prospective patient to be matched with these virtual aggregates to predict outcomes at any time point during the preoperative, perioperative and postoperative phases.

**Figure 1:**
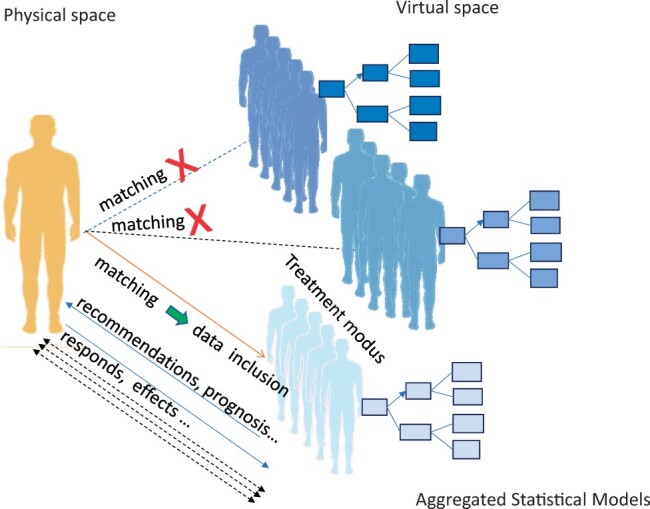
Statistical twin concept.

We define the statistical twin (as illustrated in Fig. 1) as a collection of aggregated data from multiple virtual patients throughout the treatment phase, selected at any chosen time point and based on specific input data from a large, continuously updated database. This method represents a transitional step away from traditional big data statistical techniques, moving towards individual deep phenotyping. By identifying distinctive patient groups based on demographic and clinical data, we can predict patients’ responses to treatment with less variability. This method enhances our ability to select the most suitable ‘individual’ treatment method. The ‘twin’ aspect emerges when we use machine learning to match a prospective, untreated patient with certain baseline characteristics to their ‘statistical twins’—larger data aggregates of patients undergoing various treatments. This system allows us to evaluate the potential trajectory of the patient should certain complications arise. The goal of this manuscript is to introduce the concept of the statistical twin and demonstrate its feasibility through the detection of distinctive aggregates of virtual patient groups, particularly focusing on the postoperative trajectory.

## METHODS

For the current retrospective study, we included the prospectively registered data of all patients with mitral valve disease from July 2009 through December 2018 who were discussed by the heart teams at the Maastricht University Medical Centre. This cohort of patients was published before for investigating the effect on overall survival of a dedicated mitral valve heart team compared to a general heart team for mitral valve disease [7]. This cohort was selected because it included all comers with mitral valve disease (primary mitral regurgitation, secondary mitral regurgitation and mitral stenosis) as well as patients who were treated surgically, through the transcatheter approach or with optimal medical therapy (OMT).

All clinically relevant variables were collected based on prospective recorded heart-team forms, medical records and prospectively collected data as described previously. In total, 1,332 patients with mitral valve disease were discussed. From this patient cohort, 43 patients were excluded because of active endocarditis and emergency operations.

Our local ethical committee waived the need for informed consent due to the observational and retrospective nature of the study (METC 15–4-065) for first cohort publication and in the framework of project of development of DT (METC 2022–3135).

###  

#### Mitral valve treatment decisions

##### Surgical treatment

Surgical mitral valve repair or replacement was performed via a sternotomy or using a minimally invasive (endoscopic port-access) approach.

##### Transcatheter intervention

In selected patients, mitral valve repair was performed on the beating heart using a transapical approach (NeoChord, NeoChord Inc., Minneapolis, MN, USA) [5]. Percutaneous treatments were performed with edge-to-edge repair (MitraClip, Evalve Inc, Menlo Park, CA, USA) or percutaneous annuloplasty (Carillon, Cardiac Dimension, Kirkland, WA, USA). No patient underwent valve-in-valve transcatheter therapy during the study period.

##### Optimal medical therapy group

Patients who did not have an indication for a mitral valve intervention based on the available guidelines [8, 9] were treated with OMT and followed up. Patients with non-OMT received guideline-directed medical therapy. Patients undergoing any other cardiac interventions (e.g. coronary artery bypass grafting) with concomitant mitral valve disease who were not deemed eligible for concomitant mitral valve surgery were allocated to the OMT group.

#### Follow-up

Follow-up was completed for all patients on 13 March 2020. The data were validated by 2 independent observers. The median follow-up time was 37 months (25th percentile 20, 75th percentile 71 months) validated by a local dedicated data quality surveillance team (Business Information Management Team).

## STATISTICAL ANALYSES

Baseline characteristics for all included patients are presented by mean and standard deviation or median and interquartile range for continuous variables and by count and percentage for categorical variables. Normality of the continuous variables was tested by visual inspection of the histograms and the Shapiro–Wilk test.

A two-step cluster analysis algorithm was used for the stratification to identify natural, data-dependent groups of patients based on the medical history parameters.

The first step of the analysis was selection of variables to identify a likely set of variables to “carve” the patient population into subgroups. The medical history parameters were analysed for their association with the treatment groups (1–surgery; 2–OMT). As the next step, selected variables were used for the two-step cluster procedure. Received clusters and their association with treatment groups and survival during observation period were evaluated using the Pearson χ^2^ test.

Survival was calculated from the final decision of the heart team until the last follow-up during the observation period (2009–2020); fixed time was an outcome parameter in this study.

To illustrate the data and the informational interaction between physical space and virtual patients, the effect of the surgical performance on the outcome in the clusters (virtual patients) was evaluated. We assumed that any deviation from the routine surgery flow (e.g. inexperienced surgeon, unexpected anatomical peculiarities, intraoperative complications) would increase the duration of the operation. Tukey’s hinges procedure was used to select cases with prolonged surgery. Tukey’s hinges were derived for 2 subgroups (classical and minimally invasive). The cases with a single procedure, 2 interventions and 3 or more interventions were evaluated in each subgroup. Any procedure that lasted longer than 75% of the time for a specific type of intervention was seen as “prolonged surgery”.

To evaluate the effect of postoperative complications on the survival of patients in the clusters, we identified the subgroup with an uncomplicated postoperative period and the subgroup with any postoperative complication.

Categorical variables were evaluated using the Pearson χ^2^ test. The results of the Fisher exact test [the exact significance (2-sided)] were reported if > 20% of the expected cell counts were less than 5. The analysis of variance test was used to compare continuous variables in clusters.

Tests were considered statistically significant at the 95% confidence interval (*P*-value ≤ 0.05). Statistical analyses were performed using SPSS version 23 (SPSS Inc., Chicago, IL, USA).

## RESULTS

### Population

Among the 1289 patients analysed, 574 (44.5%) underwent surgical treatment; 108 (8.4%) had a transcatheter intervention; and 607 (47.1%) were treated with OMT (Table 1). There were 593 (46%) females. The mean age of the patients in the study was 69.5 (SD: 10.9) years. Of the patients included in the study, 910 (70.6%) survived for the 10-year observation period (2009–2019). In the surgical treatment group, 453 (74.6%) patients and 373 (61.4%) patients treated with OMT were alive at the end of the observation period. There were no missing data, and all the analyses were done with the full data set.

**Table 1:** Association of medical history parameters with the treatment group* (sample size = 1289).

Parameter	Degrees of freedom	χ^2^ statistical value	*P*-value
Extracardiac arteriopathy	2	3.209	0.201
Poor mobility	2	1.344	0.511
Previous cardiac surgery	2	13.670	0.001
Chronic lung disease	2	8.438	0.015
Diabetes on insulin	2	9.858	0.007
NYHA class	6	12.998	0.043
Left ventricle function	6	70.116	<0.001
Recent myocardial infarction	2	13.019	0.001
Pulmonary hypertension	4	133.752	<0.001
Renal function	6	206.786	<0.001

* 1–surgical treatment; 2–conservative (optimal medical treatment).

The medical history variables used in this study are those used by EuroSCORE II. Table 1 depicts the association of the medical history parameters with the treatment group (1–surgery; 2–OMT). Based on the *P*-values and the opinions of the experts, 5 parameters (renal function, previous cardiac surgery, diabetes on insulin, left ventricle function, pulmonary hypertension) were selected for the cluster procedure.

### Cluster analysis at baseline

**Figure 2:**
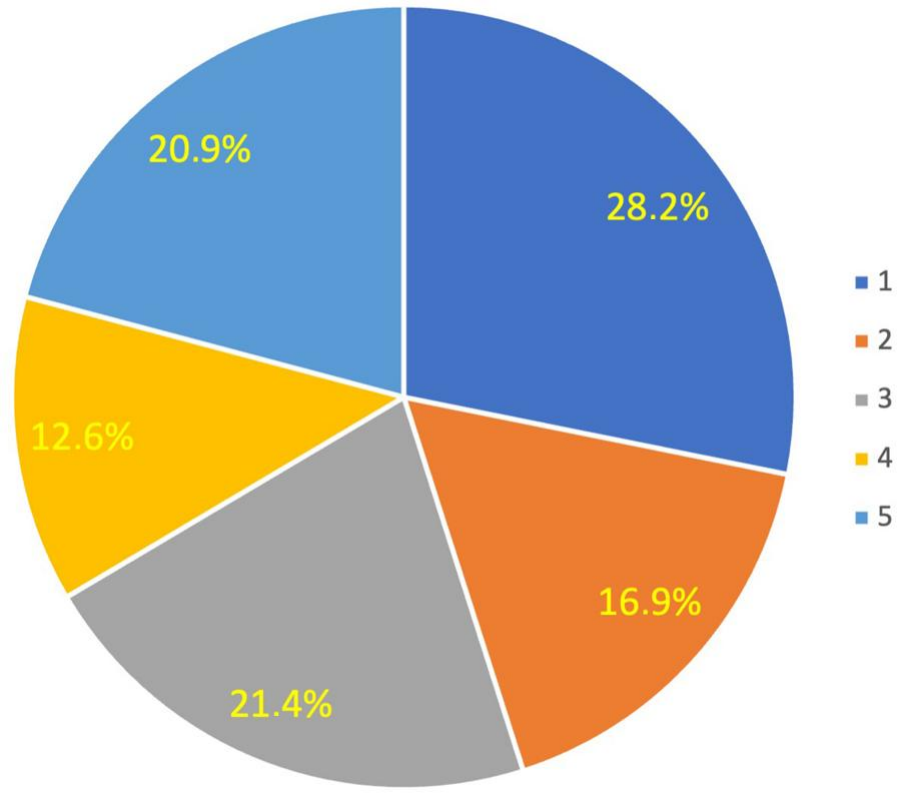
Distribution of patients in clusters.

**Figure 3:**
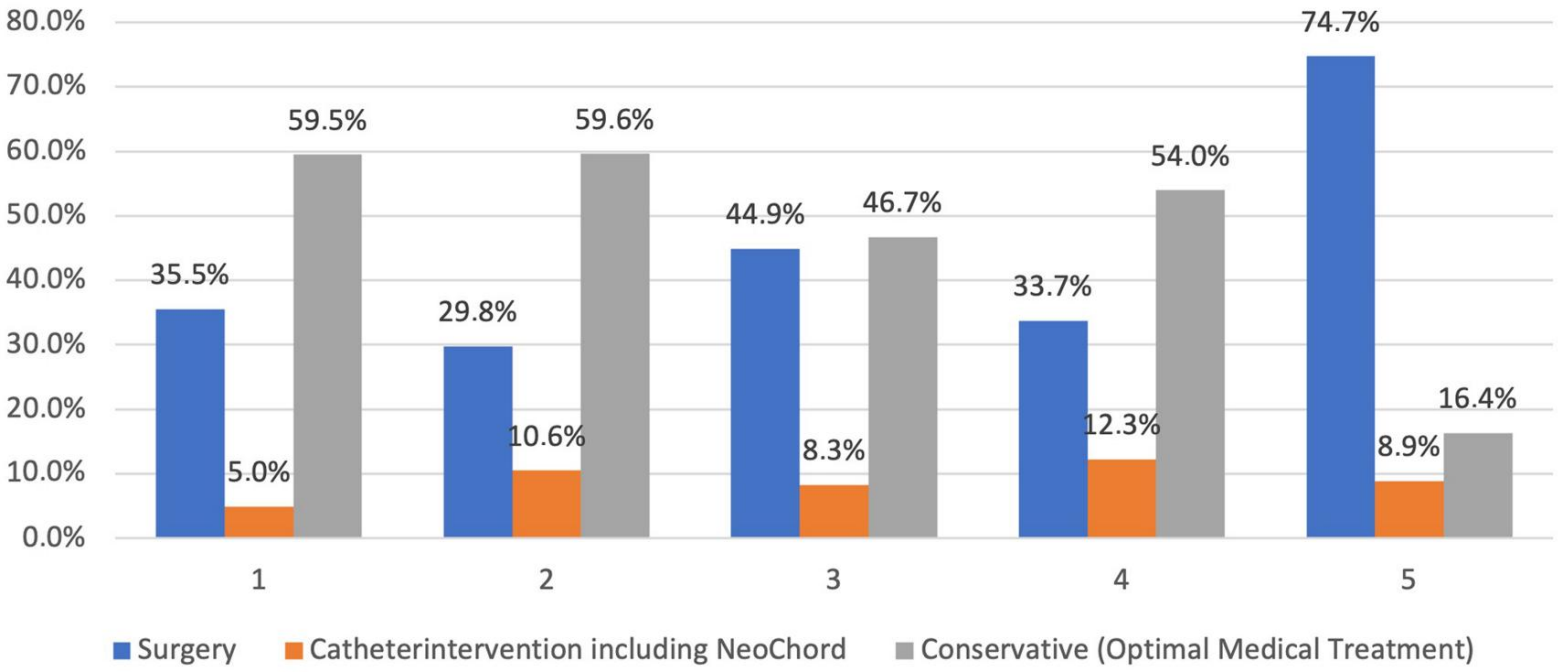
Treatment groups in clusters: χ^2^ (degree of freedom = 8, sample size = 1289, *P* < 0.001).

The cluster procedure resulted in the selection of 5 distant clusters (average silhouette = 0.6) with relatively equal numbers of patients (Fig. 2). The distribution of the medical history parameters is shown in the clusters in Tables 1S–5S. The distribution of patients by treatment groups (surgical, transcatheter intervention and OMT) within the clusters revealed 2 clusters with the prevalence of patients treated with OMT (clusters 1 and 2), 2 mixed clusters (clusters 3 and 4) and 1 cluster with more patients treated surgically (cluster 5) (Fig. 3).

**Figure 4:**
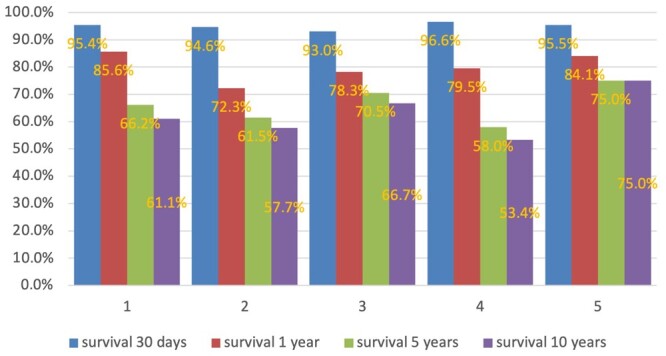
Survival rate after the final decision of the heart team in the case of optimal medical therapy. Only 1-year survival was different in clusters: χ^2^ (degree of freedom = 4, sample size = 607, *P* = 0.042).

**Figure 5:**
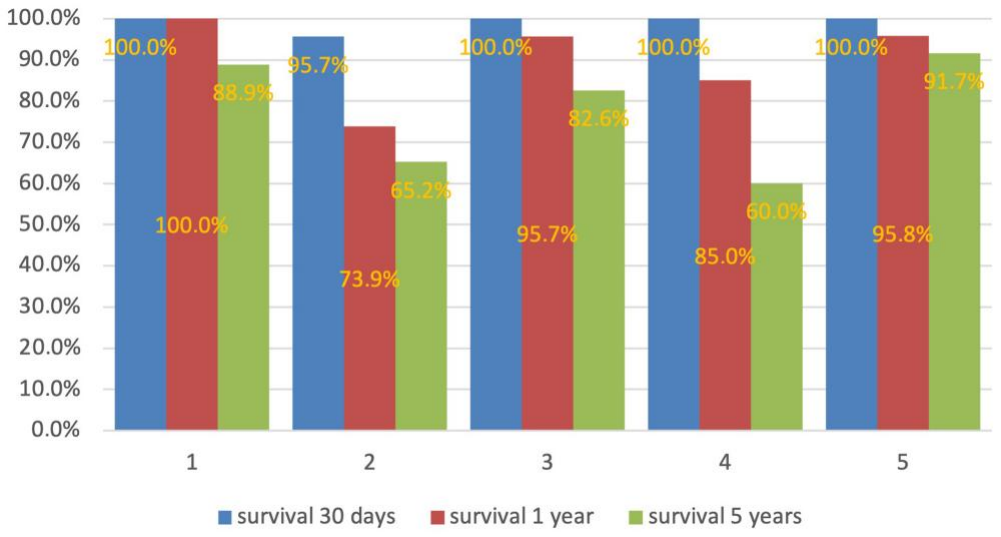
Survival rate after the final decision of the heart team in the case of transcatheter intervention including the NeoChord. One-year [χ^2^ (degree of freedom = 4, sample size = 108, *P* = 0.03)] and 5-year [χ^2^(degree of freedom 4, sample size = 108, *P* = 0.04)] survival rates were dependant on the characteristics of the virtual patients. Maximum observation period was 5 years.

In virtual patients treated surgically, the EuroSCORE II values were significantly different (Table 6S, appendix, F (4,569) = 71.45, *P* < 0.001). However, we did not find any relation between the actual 30-day postoperative mortality and the EuroSCORE II (Fig. 1S; Pearson correlation coefficient = 0.149, *P* = 0.811) in our set of clusters (virtual patients). The statistical model in this example demonstrated that in cases with a decision in favour of OMT, the expected survival rate changed from 95–96% in 30 days to 58–75% in 10 years (Fig. 4). The changes in survival rate were not affected by the characteristics of the virtual patients in our example. In the case of transcatheter interventions, including the NeoChord, the survival rate after the heart team’s final decision at 5 years was 60–92% (Fig. 5). Furthermore, 1 year [*χ*^2^ (degree of freedom = 4, sample size = 108, *P*-value = 0.03) and 5-year [*χ*^2^ (degree of freedom = 4, sample size = 108, *P*-value = 0.04)] survival rates were dependant on the characteristics of the virtual patients.

### Inclusion of perioperative factors and influence on prognosis

**Figure 6:**
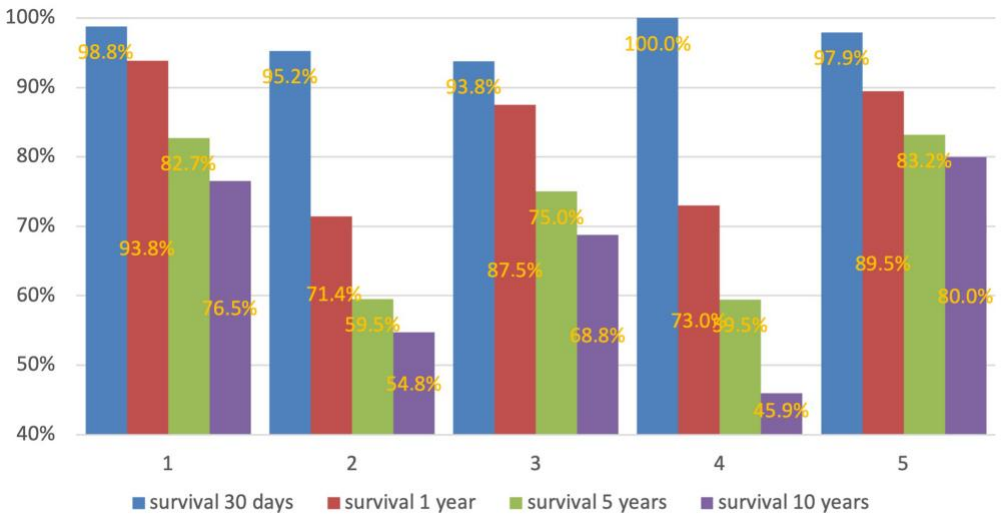
Survival rate after the final decision of the heart team in the case of surgical treatment. (a) Cases with normal surgery duration and uncomplicated postoperative period. Survival rates at 1 [χ^2^ (degree of freedom = 4, sample size = 498, *P* = 0.012), 5 [χ^2^ (degree of freedom 4, sample size = 498), *P* < 0.001), and 10 years [χ^2^ (degree of freedom = 4, sample size = 498), *P* < 0.001)] were dependant on the characteristic of the virtual patients; (b) cases with extended surgery duration and/or with a complicated postoperative period. Survival rates at 1 [χ^2^ (degree of freedom = 4, sample size = 319), *P* = 0.001)], 5 [χ^2^ (degree of freedom = 4, sample size = 319, *P* = 0.003)], and 10 years [χ^2^ (degree of freedom = 4, sample size = 319, *P* < 0.001)] were dependant on the characteristics of the virtual patients.

Another illustration of the two-way communication of physical and virtual patients is the dynamic change in prognosis when the aggregated statistical model includes information about perioperative factors. In this example, the survival rate of cases with uncomplicated surgical treatment of normal duration was higher throughout the entire observation period in comparision to the statistical model, which included perioperative complications and extended surgical durations beyond the 75th percentile (Fig. 6; Figs. 2S and 3S; Tables 7S and 8S).

**Figure 7:**
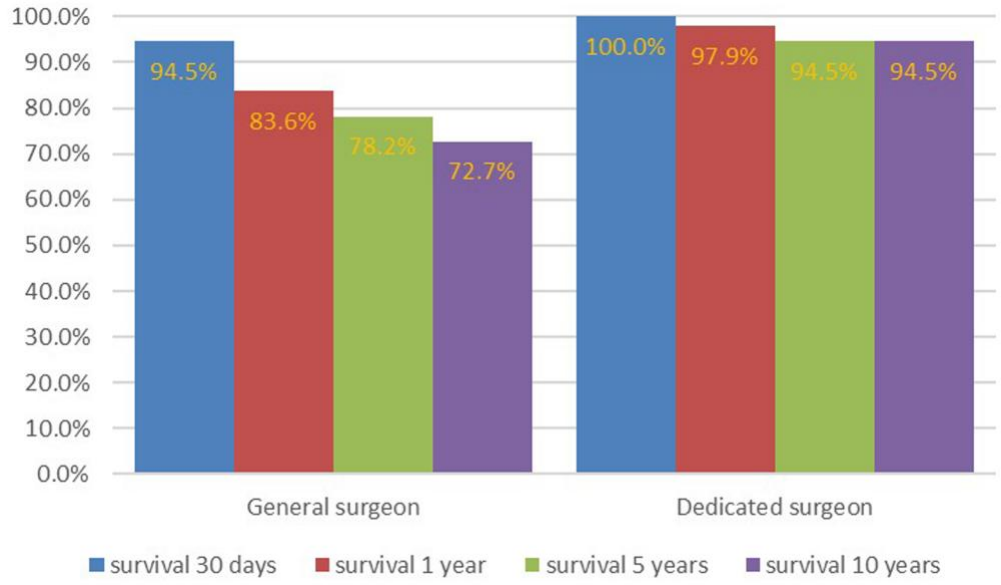
Effect of surgical team experience on survival of aggregated virtual patient cluster 5. Survival rates at 30 days [χ^2^ (degree of freedom = 1, sample size = 201, *P* = 0.004)]; survival at 1 year [χ^2^ (degree of freedom = 1, sample size = 201, *P* < 0.001)]; survival at 5 years [χ^2^ (degree of freedom = 1, sample size = 201, *P* < 0.001)]; survival at 10 years [χ^2^ (degree of freedom = 1; sample size = 201, *P* < 0.001)].

The influence of external factors on the virtual model is shown in Fig. 7. The aggregated virtual patients of cluster 5 would have had better survival rates at all time points if the interventions were done by a dedicated surgeon.

## DISCUSSION

We have introduced the concept of statistical twin as a transition to full digital twin technology. We define statistical twin as larger aggregates of the data of multiple patients throughout the treatment phase at any chosen time point based on chosen input data. The prerequisite for using statistical twin is the ability to detect distinct aggregates of the data of multiple patients at multiple time points. We show that this goal is conceptually possible, even when it is based on a limited number of variables.

Statistical twin is driven by the concept of digital twin. In health care, the ‘digital twin’ denotes a comprehensive, virtual tool that integrates the clinical data acquired over time for an individual using mechanistic and statistical models. Mechanistic models represent our knowledge of the physiology and the laws of physics and chemistry. These models provide a framework in which to integrate and augment the experimental and the clinical data, enabling the identification of the mechanisms and/or the prediction of outcomes. In a complementary way, statistical models represent the knowledge and relations induced from the data. They allow the extraction and optimal combination of individualized characteristics using mathematical rules. Synergy between mechanistic and statistical models is the basis of digital twin models in cardiovascular medicine [10].

There are many misconceptions about digital twin, specifically about “*How to tell the difference between a model and a digital twin*”. The prerequisites of a digital twin are having a **model** of the object; an **evolving set of data** related to the object and a **means of dynamically updating or adjusting the model** in accordance with the data [11].

The development of any statistical model is subjected to many cofounders that are non-correctable from the time frame of the studies, the institutional variations, the individual variations and the development and implementation of new treatments. The advantage of statistical twin is that we can develop a general framework of the statistical twin modelling system (e.g. general input data, time points of aggregates) that is validated in different data sets, and we can use this framework at the institutional or regional level and update it continuously using local data sets. This procedure allows for the continuous correction of institutional variations, individual variations, new developments and implementation of new treatments. The development of machine learning algorithms in combination with statistical twin modelling allows us to find the twins of a prospective patient for whom different treatment modalities are possible to facilitate decision making. We can offer various scenarios for different therapeutic strategies, considering the potential major complications and their impact on the outcomes when we make heart-team decisions. In addition, for a prospective patient undergoing a treatment, statistical twin modelling informs the caregivers during the process of care about patients at risk for the need of adjustment of their treatment trajectory. Furthermore, postoperative treatment is increasingly dependent on multidisciplinary care teams, so continuously updated risk models could present an opportunity for personalized perioperative care [12].

Beginning in the 1980s with the Parsonnet score, the centre of attention of risk models was originally the pre-operative prediction of death. At least 19 risk-stratification models existed for open-heart surgery [13]. These models were derived from studies of large populations and could be very effective at predicting population outcomes but are not necessarily suited for the prediction of the risk of an individual patient [14]. Furthermore, despite sporadic publication of articles underlining the importance of perioperative events for the prediction of outcome, these variables are not taken into account in risk score systems [13–15].

We limited our example to the demonstration of the dependence of the outcome on the preoperative condition and the perioperative and external factors. However, in contrast to the risk models, the goal of the statistical twin is not only to predict outcomes but also to help in selecting patients’ optimal treatment trajectory.

**Figure 8:**
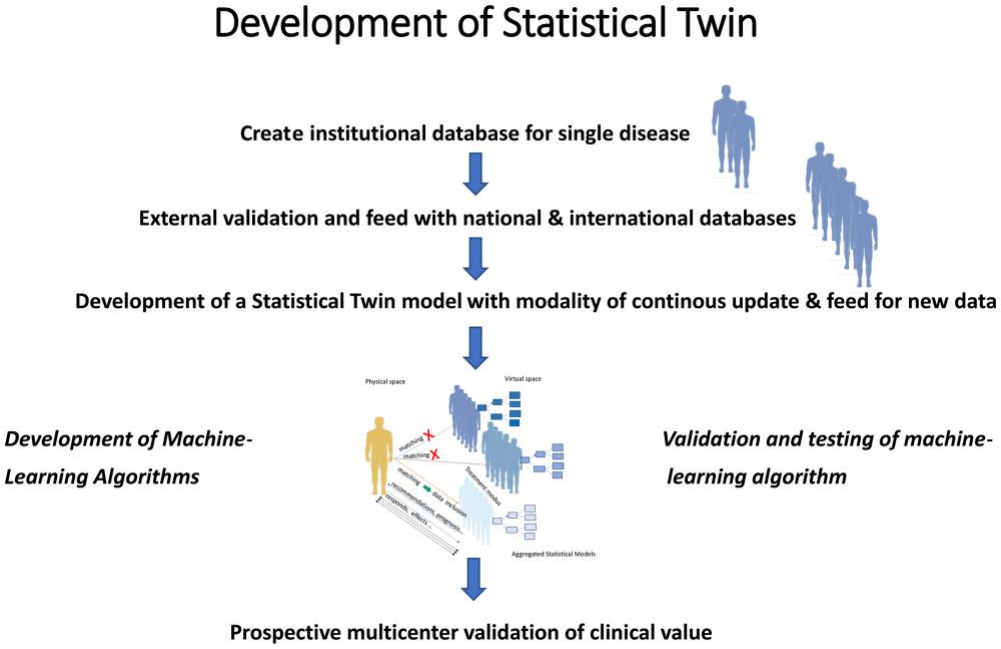
Development of statistical twin.

We introduced the concept of statistical twin and provided the proof of concept of its prerequisite, namely, the ability to detect distinct aggregates of data from multiple patients at multiple time points. For further development of statistical twin, we propose the following trajectory (Fig. 8): creation and development of a large database for modelling for a single disease at the institutional level; external validation using a national database; development of statistical twin modelling for continuous data input at the institutional level; development of a machine learning algorithm; validation of a machine learning algorithm; testing algorithms; and prospective multicentre validation.

We hope that this example explains the concept of statistical twin. However, the virtual patient model we introduce lacks some important characteristics that are necessary for the ‘twin’ model. First, we included a limited number of parameters in the cluster analysis. However, including maximum available as well as unavailable but required attributes in the creation of ‘virtual patients’ calls for special methods of cluster analysis of high-dimensional data [16] and much larger and more complete databases. Also, due to the limited number of patients in the database, we did not analyse the influences of the valve condition, intracardiac haemodynamics, method of surgical intervention (classical or minimized), regular outliers and extreme outliers of surgical duration, effects of postoperative complications on hospitalization and outcomes. Also, the development of specific postoperative complications and their relation to the preoperative condition and intraoperative events were not evaluated. We did not include multiple external factors (e.g. virtual models of surgeons, surgical and intensive care unit teams with two-way communication with their physical space subjects). Development of a statistical twin model that could communicate with external virtual models would improve its quality [6].

## CONCLUSION

We introduced the concept of statistical twin as several aggregated virtual patients. The demographic and clinical data from these aggregated virtual patients were matched with the data from the physical entity (real patient). The real patient with the closest match became the representation of or the model of a specific patient in cyberspace. In a completely developed statistical twin, two-way communication between the physical entity and the digital counterpart enables the model to validate, optimize, evaluate, diagnose the real part and suggest predictions based on data analysis throughout the treatment trajectory. Further development and validation of the concept of statistical twin are needed to unlock its potentials.

## Supplementary Material

ivae032_Supplementary_Data

## Data Availability

The data will be shared upon reasonable request from the corresponding author.

## References

[1] Nashef SA , Roques F, Michel P, Gauducheau E, Lemeshow S, Salamon R. European system for cardiac operative risk evaluation (EuroSCORE). Eur J Cardiothorac Surg 1999:9–13. doi: 10.1016/s1010-7940(99)00134-7. 10456395

[2] Shahian DM , O'Brien SM, Filardo G, Ferraris VA, Haan CK, Rich JB, Society of Thoracic Surgeons Quality Measurement Task Force et al The Society of Thoracic Surgeons 2008 cardiac surgery risk models: part 1—coronary artery bypass grafting surgery. Ann Thorac Surg 2009;88:S2–22. doi: 10.1016/j.athoracsur.2009.05.053. 19559822

[3] Björnsson B , Borrebaeck C, Elander N, Gasslander T, Gawel DR, Gustafsson M, ; Swedish Digital Twin Consortium et al Digital twins to personalize medicine. Genome Med 2019;12:4. doi: 10.1186/s13073-019-0701-3. 31892363 PMC6938608

[4] Venkatesh KP , Raza MM, Kvedar JC. Health digital twins as tools for precision medicine: considerations for computation, implementation, and regulation. NPJ Digit Med 2022;5:150. doi: 10.1038/s41746-022-00694-7. 36138125 PMC9500019

[5] Coorey G , Figtree GA, Fletcher DF, Snelson VJ, Vernon ST, Winlaw D et al The health digital twin to tackle cardiovascular disease-a review of an emerging interdisciplinary field. NPJ Digit Med 2022;5:126. doi: 10.1038/s41746-022-00640-7. 36028526 PMC9418270

[6] Shengli W. Is Human Digital Twin possible? Computer Methods and Programs in Biomedicine Update 2021;1:100014.

[7] Sardari Nia P , Olsthoorn JR, Heuts S, van Kuijk SMJ, Vainer J, Streukens S et al Effect of a dedicated mitral heart team compared to a general heart team on survival: a retrospective, comparative, non-randomized interventional cohort study based on prospectively registered data. Eur J Cardiothorac Surg 2021;60:263–73. doi: 10.1093/ejcts/ezab065. 33783480

[8] Nishimura RA , Otto CM, Bonow RO, Carabello BA, Erwin JP, Fleisher LA et al 2017 AHA/ACC Focused Update of the 2014 AHA/ACC Guideline for the Management of Patients With Valvular Heart Disease: a Report of the American College of Cardiology/American Heart Association Task Force on Clinical Practice Guidelines. J Am Coll Cardiol 2017;70:252–89. 28315732 10.1016/j.jacc.2017.03.011

[9] Baumgartner H , Falk V, Bax JJ, De Bonis M, Hamm C, Holm PJ, ESC Scientific Document Group et al 2017 ESC/EACTS Guidelines for the management of valvular heart disease. Eur Heart J 2017;38:2739–91. 28886619 10.1093/eurheartj/ehx391

[10] Corral-Acero J , Margara F, Marciniak M, Rodero C, Loncaric F, Feng Y et al The ‘Digital Twin’ to enable the vision of precision cardiology. Eur Heart J 2020;41:4556–64. doi: 10.1093. 32128588 10.1093/eurheartj/ehaa159 PMC7774470

[11] Wright L , Davidson S. How to tell the difference between a model and a digital twin. Adv Model and Simul in Eng Sci 2020;7:13. 10.1186/s40323-020-00147-4.

[12] Durant TJS , Jean RA, Huang C, Coppi A, Schulz WL, Geirsson A et al Evaluation of a Risk Stratification Model Using Preoperative and Intraoperative Data for Major Morbidity or Mortality After Cardiac Surgical Treatment. JAMA Netw Open 2020;3:e2028361. doi: 10.1001/jamanetworkopen.2020.28361. 33284333 PMC11841993

[13] Prins C , de Villiers Jonker I, Botes L, Smit FE. Cardiac surgery risk-stratification models. Cardiovasc J Afr 2012:160–4. doi: 10.5830/CVJA-2011-047. 22555640 PMC3721858

[14] Granton J , Cheng D. Risk stratification models for cardiac surgery. Semin Cardiothorac Vasc Anesth 2008:167–74. doi: 10.1177/1089253208323681. 18805851

[15] Stoica SC , Sharples LD, Ahmed I, Roques F, Large SR, Nashef SA. Preoperative risk prediction and intraoperative events in cardiac surgery. Eur J Cardiothorac Surg 2002:41–6. doi: 10.1016/s1010-7940(01)01077-6. 11788254

[16] Han J et al (2011). Data Mining: Concepts and Techniques. San Francisco, CA, USA, Morgan Kaufmann Publishers Inc.

