# Supplementary material for: Proposal of statistical twin as a transition to full digital twin technology for cardiovascular interventions
Source: Interdiscip Cardiovasc Thorac Surg. 2024 Mar 4;38(4):ivae032. doi: 10.1093/icvts/ivae032 (PMC11001486; doi:10.1093/icvts/ivae032)
Supplement: ivae032_Supplementary_Data [file ivae032_supplementary_data.docx]

**Supplementary files to: Proposal of Statistical Twin as transition to full Digital Twin technology for cardiovascular interventions.**

Table 1S. Renal function in clusters*

| Renal function | TwoStep Cluster Number | | | | | Total |
| --- | --- | --- | --- | --- | --- | --- |
|  | 1 | 2 | 3 | 4 | 5 |  |
| Normal clearance GFR > 80 | 363 | 130 | 178 | 101 | 36 | 808 |
| Mildly decreased GFR > 50 < 84 | 0 | 49 | 69 | 34 | 169 | 321 |
| Severely decreased GFR < 50 | 0 | 37 | 29 | 28 | 64 | 158 |
| Dialysis | 0 | 2 | 0 | 0 | 0 | 2 |
| Total | 363 | 218 | 276 | 163 | 269 | 1289 |

* *Chi-squared* (degree of freedom 12, sample size= 1289), p < 0.001.

Table 2S. Earlier cardiac intervention.*

|  |  | TwoStep Cluster Number | | |  |  | Total |
| --- | --- | --- | --- | --- | --- | --- | --- |
|  |  | 1 | 2 | 3 | 4 | 5 |  |
| Earlier cardiac intervention | No | 363 | 208 | 276 | 0 | 269 | 1116 |
|  | Yes | 0 | 10 | 0 | 163 | 0 | 173 |
| Total |  | 363 | 218 | 276 | 163 | 269 | 1289 |

**Chi-squared* (degree of freedom 4, sample size = 1289), p < 0.001.

Table 3S. Insulin dependent diabetes.*

|  |  | 1 | 2 | 3 | 4 | 5 | Total |
| --- | --- | --- | --- | --- | --- | --- | --- |
| Insulin dependent diabetes | No | 363 | 178 | 274 | 162 | 269 | 1246 |
|  | Yes | 0 | 40 | 2 | 1 | 0 | 43 |
| Total |  | 363 | 218 | 276 | 163 | 269 | 1289 |

* *Chi-squared* (degree of freedom 4, sample size = 1289), p < 0.001.

Table 4S. Left ventricle function.*

| Ejection fraction | TwoStep Cluster Number | | | | | Total |
| --- | --- | --- | --- | --- | --- | --- |
|  | 1 | 2 | 3 | 4 | 5 |  |
| Normal (> 50%) | 363 | 43 | 0 | 75 | 269 | 750 |
| Mildly reduced (31-50%) | 0 | 29 | 276 | 66 | 0 | 371 |
| Reduced (21-30%) | 0 | 103 | 0 | 16 | 0 | 119 |
| Severely reduced (<21%) | 0 | 43 | 0 | 6 | 0 | 49 |
| Total | 363 | 218 | 276 | 163 | 269 | 1289 |

**Chi-squared* ( degree of freedom 12, sample size = 1289),.3, p < 0.001.

Table 5S. Pulmonary hypertension.*

| Pulmonary hypertension | TwoStep Cluster Number | | |  |  | Total |
| --- | --- | --- | --- | --- | --- | --- |
|  | 1 | 2 | 3 | 4 | 5 |  |
| No | 363 | 155 | 224 | 129 | 151 | 1022 |
| Mild (PP 31-55 mmHg) | 0 | 15 | 52 | 25 | 118 | 210 |
| Severe (PP >55 mmHg) | 0 | 48 | 0 | 9 | 0 | 57 |
| Total | 363 | 218 | 276 | 163 | 269 | 1289 |

**Chi-squared* (degree of freedom12, sample size= 1289), p < 0.001.

Table 6S**.** Euroscore II in clusters*

| Cluster | N | Mean | Std. Deviation |
| --- | --- | --- | --- |
| 1 | 129 | 1.2 | 0.9 |
| 2 | 65 | 6.3 | 5.4 |
| 3 | 124 | 3.5 | 2.4 |
| 4 | 55 | 7.0 | 4.2 |
| 5 | 201 | 2.3 | 1.7 |
| Total | 574 | 3.2 | 3.3 |

*F (4,569) = 71.45, p<0.001

|  |
| --- |
| **Figure 1S.** 30 days mortality and ES II in clusters. Pearson correlation coefficient= 0.149, p = 0.811 |

| 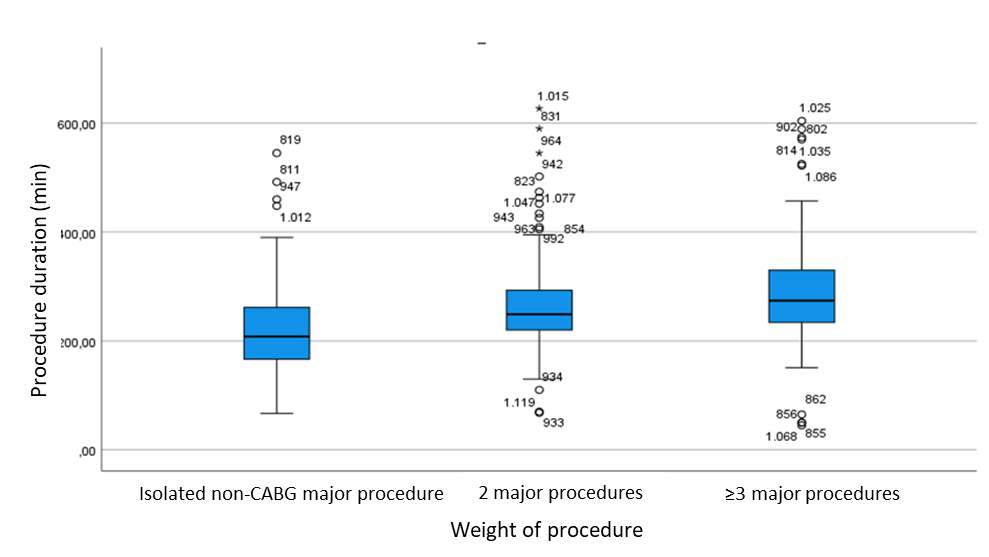 |
| --- |
| Figure 2S. Distribution of classical interventions by its duration. The upper hinge in single non-CABG intervention was 261.5 minutes. in 2- interventions – 293 minutes. and in 3 and more interventions – 330 minutes. |

| 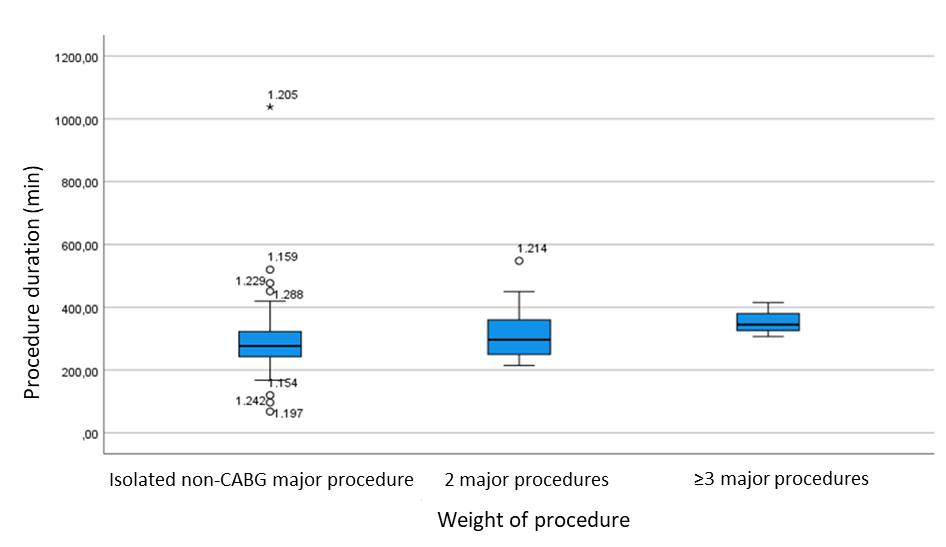 |
| --- |
| Figure 3S. Distribution of minimal invasive interventions by its duration. The upper hinge in single non-CABG intervention was 323 minutes. in 2- interventions – 360 minutes. and in 3 and more interventions – 380 minutes. |

Table 7S. Mortality rate by cluster in patients with normal surgery duration and with uncomplicated postoperative period. *

|  | | TwoStep Cluster Number | | | | | Total |
| --- | --- | --- | --- | --- | --- | --- | --- |
|  |  | 1 | 2 | 3 | 4 | 5 |  |
| Dead | No | 28 | 9 | 32 | 12 | 54 | 135 |
|  | Yes | 3 | 6 | 5 | 0 | 3 | 17 |
| Total |  | 31 | 15 | 37 | 12 | 57 | 152 |

* *chi-squared* (degree of freedom 4. Sample size = 152), p = 0.003.

Table 8S. Mortality rate by cluster in patients with extended surgery duration and/or with complicated postoperative period.*

|  | | TwoStep Cluster Number | | | | | Total |
| --- | --- | --- | --- | --- | --- | --- | --- |
|  |  | 1 | 2 | 3 | 4 | 5 |  |
| Dead | No | 62 | 23 | 44 | 17 | 76 | 222 |
|  | Yes | 19 | 19 | 20 | 20 | 19 | 97 |
| Total |  | 81 | 42 | 64 | 37 | 95 | 319 |

**chi-squared* (degree of freedom 4. Sample size = 319), p <0.001.
